# Patterns of myeloarchitecture in lower limb amputees: an MRI study

**DOI:** 10.3389/fnins.2015.00015

**Published:** 2015-02-05

**Authors:** Eyesha Hashim, Christopher D. Rowley, Sharon Grad, Nicholas A. Bock

**Affiliations:** ^1^Medical Physics and Applied Radiation Sciences, McMaster UniversityHamilton, ON, Canada; ^2^Physical Medicine and Rehabilitation, McMaster UniversityHamilton, ON, Canada

**Keywords:** primary motor cortex, intracortical myelin, amputation, magnetic resonance imaging, cortical morphology

## Abstract

Functional studies of cortical plasticity in humans suggest that the motor cortex reorganizes when the descending motor output pathway is disrupted as a result of limb amputation. The question thus arises if the underlying anatomical organization of the motor cortex is also altered in limb amputation. Owing to challenges involved in imaging the thin cerebral cortex *in vivo*, there is limited data available on the anatomical or morphological plasticity of the motor cortex in amputation. In this paper, we study the morphology of the primary motor cortex in four lower limb amputees with 37 or more years of amputation and four age and gender-matched controls using 0.7 mm isotropic, T_1_-weighted MRI optimized to produce enhanced intracortical contrast based on myelin content. We segment the cortex into myelinated and unmyelinated gray matter. We determine the myelinated thickness which is the thickness of the well-myelinated tissue in the deeper layers of the cortex. We compare the bilateral differences in the myelinated thickness between amputees and controls. We also compare bilateral differences in cortical thickness between the two groups. Our measurements show no statistically significant difference between the amputees and controls in the myelinated thickness and in cortical thickness, in the region of the primary motor cortex representing the lower leg.

## Introduction

Functional plasticity studies in the human cortex document reorganization in primary regions as a consequence of major changes in function (Rockstroh et al., 2000; Qi et al., 2010; Roiha et al., 2011). In upper limb amputees, for example, the face and stump muscle representations in the primary motor cortex (M1) have been shown to be displaced toward the former hand representation (Lotze et al., 2001). Such evidence of functional reorganization in the presence of disrupted motor output pathways suggests that there may be accompanying changes in the structure of the primary motor cortex. With recent advances in neuro-anatomical magnetic resonance imaging (MRI) and image processing techniques, it is now possible to investigate the morphological features of cortical areas associated with primary functions, such as M1, for plasticity studies.

In this paper, we use T_1_ -weighted anatomical MRI to investigate if the anatomical structure of the primary motor cortex in the pre-central gyrus is grossly altered as a result of lower limb amputation in humans. Specifically, we image intracortical myelin, whose distribution can delineate motor regions in the human cortex on MRI (Bock et al., 2013; Stuber et al., 2014).

In a basic description of the motor system, upper motor neurons in the motor cortex synapse with lower motor neurons in the spinal cord which then project to muscles. Limb amputation severs the axons of the lower motor neurons that were innervating the now lost limb, thereby disrupting that motor pathway. The question then arises if this disruption in the motor pathway affects cortical structure. For instance, the upper motor neurons and their axons that were contributing to the movement of muscles in the previously intact limb could degenerate when their prescribed output pathway is disrupted. As the descending axons of the upper motor neurons are well myelinated (Bishop and Smith, 1964), their degeneration would result in loss of intracortical myelin in the contralateral motor cortex and would thus result in bilateral asymmetry in the intracortical myelin pattern in amputees. Alternatively, these upper motor neurons may remain viable with their myelinated axons intact, even if they have rewired to innervate other body areas. In this case, no bilateral asymmetry in the intracortical myelin pattern in the amputees would be expected.

A degeneration of upper motor neurons and their associated myelinated axons could affect several structural features in the motor cortex including overall gray matter volume and the thickness of the cortical tissue. The available data on structural changes in the brain resulting from the loss of motor output is limited and contradictory. For example, one volumetric study in amputees reported a loss of gray matter in M1 (Preissler et al., 2013), while another study did not find any such loss (Draganski et al., 2006).

In this study, we investigate the thickness of the deeper myelinated layers in the cortex to assess changes in intracortical myelin in the lower leg representation in the motor homunculus of amputees. The thickness of the deeper myelinated layers in the cortex has been used in studies of schizophrenia to estimate changes in intracortical myelin (Bartzokis and Altshuler, 2005). We also investigate cortical thickness in the lower leg representation in the motor homunculus of amputees to identify changes in cortical morphology as a result of long withstanding amputation.

We image the cortex using a T_1_-weighted MRI sequence at 3T, optimized to obtain enhanced contrast across the cortex based on intracortical myelin (Bock et al., 2013). The primary motor cortex is the thickest part of the cortex at 4.5 mm (Zilles and Amunts, 2012) and we image it using 0.7 mm isotropic resolution to improve visualization of intracortical myelin vs. a typical anatomical MRI at 1 mm isotropic resolution. The Brodmann area 4 (BA 4) is considered to be the anatomical analog of the functionally defined primary motor cortex which covers the anterior wall of the central sulcus, the dorso-medial part of the free surface of the pre-central gyrus and approximately middle one-third of the para-central lobule in the medial wall of the hemisphere (Rizzolatti et al., 1998; Matelli et al., 2004). Studies of myeloarchitecture indicate that M1 has a unique myelination pattern with the strongly myelinated bands of Baillarger (Baillarger, 1840) merging together which results in continous heavy myelination through the deeper cortical layers to the white matter. (Vogt and Vogt, 1919; Vogt, 1951; Braitenberg, 1962; Braak, 1980; Nieuwenhuys, 2013). The radial bundles of myelinated fibers in M1 further extend beyond the outer stripe of Baillarger to the deeper parts of the upper pyramidal layer, which means that heavy myelination in M1 persists continuously through most of the cortical thickness in this area. We therefore propose to use the thickness of the well-myelinated tissue in M1 as a metric to investigate structural plasticity in lower limb amputees. We refer to the thickness of the well-myelinated tissue as the myelinated thickness (*m*). We segment the brain tissue into well-myelinated cortical gray matter (mGM), relatively unmyelinated cortical gray matter (GM), white matter (WM) and cerebrospinal fluid (CSF) and define the myelinated thickness as the distance between the outer boundaries of mGM and WM.

Functional studies of the homunculus in humans indicate that the upper motor neurons which project to the lower motor neurons innervating the lower limbs are located in the dorso-medial part of M1, with those in the medial wall of the gyrus contributing to the movement of the part of the leg below the knee (Penfield and Rasmussen, 1950). Therefore, we expect that if structural degeneration of these upper motor neurons happens as a consequence of lower limb amputation, a decrease in the myelinated thickness in this part of the contralateral M1 could be observed. There could also be a corresponding reduction in the total cortical thickness (*t*). We thus measure *m* and *t* in the area representing the lower leg in M1 in both hemispheres in four lower limb amputees and in their age and sex matched healthy controls to investigate the bilateral differences in these parameters. Through these comparisons, we hope to gain insight if upper motor neurons degenerate when they are deprived from their original output, resulting in bilateral changes in the morphology of the primary motor cortex.

## Methods

### Subjects

The subjects for this study were four male, lower-limb amputees and four male age-matched controls. None of the subjects reported neurological conditions. The nature of the injuries in the amputees is summarized in Table 1. The study was approved by the Research Ethics Board at Saint Joseph's Hospital and informed consent was obtained from all subjects prior to imaging.

**Table 1:** **Subject information**.

	**Age of amputee (years)**	**Lost limb**	**Duration of loss (years)**	**Age of matched control (years)**
1	46	Left leg; below knee	37	44
2	51	Right leg; below knee	49	50
3	72	Left leg; below knee	65	64
4	49	Right leg; below knee, above knee	39, 4	50

### MRI

Subjects were scanned on a 3 Tesla General Electric (GE) scanner (Software Version DV 22.0) with a whole body transmit coil (GE) and a 32 channel receive coil (MR Instruments). The details of the imaging protocols are summarized in Table 2. A 3D, T_1_-weighted inversion recovery, gradient echo sequence (BRAVO) was optimized to obtain enhanced intracortical contrast, suitable for visualizing intracortical myelin at 0.7 mm isotropic resolution (Bock et al., 2013). To keep the imaging time as short as possible in light of the high resolution, partial brain images were acquired with an 8 cm wide, rectangular slab, approximately centered at the pre-central gyrus along the anterior-posterior axis. The slab fully encompassed the central sulcus laterally. The field-of-view (FOV) covered the brain fully along the other two anatomical axes. The T_1_-weighted BRAVO scan was followed by a predominantly proton density-weighted, 3D FLASH (Fast, Low Angle Shot) acquisition over the same FOV, to be used in post-processing to compensate for RF field inhomogeneities (Van de Moortele et al., 2009; Marques et al., 2010). The total acquisition time for the protocol was about 40 min per subject.

**Table 2:** **A summary of the imaging parameters**.

**Parameter**	**Optimized BRAVO**	**FLASH**
Excitation angle α	12°	4°
FOV_readout_ × FOV_*pe1*_ × FOV_*pe2*_	240 × 192 × 82.6 (mm)	240 × 192 × 82.6 (mm)
Imaging matrix	344 × 275 × 118	344 × 275 × 118
Number of α pulses per shot (*N*_pe2_)	118	118
Resolution	0.7 mm isotropic	0.7 mm isotropic
*TR* (between successive α pulses)	9.9 ms	9.9 ms
*TE* (echo time)	4.1 ms	4.1 ms
*TI* (inversion time)	1000 ms	–
*TD* (time delay between consecutive inversions)	1100 ms	–
Acquisition time	15 min	5.3 min
Repeats	2	2

*N stands for the number of excitation pulses; pe1 represents the first phase encoding direction while pe2 is the second phase encoding direction*.

### Post processing

**Figure 1:**
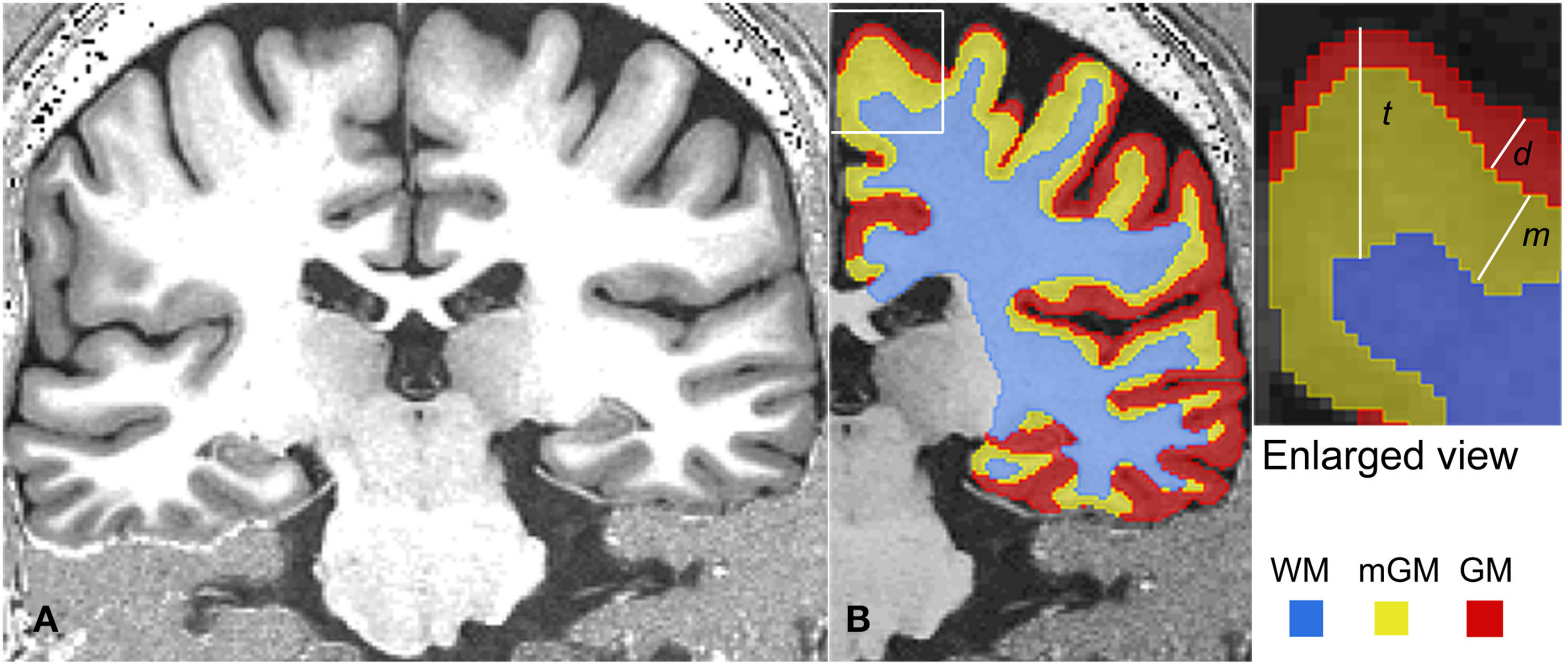
**A representative MRI slice is shown in (A)**. The tissue classification for the right hemisphere of this MRI is shown in **(B)**. On the right, various thickness measurements are shown schematically in an enlarged corner of **(B)**. Here, cortical thickness is represented as *t*, the thickness of the myelinated cortex as *m* and depth of the myelinated cortex below the outer cortical boundary as *d*.

To minimize postural differences during image acquisition, the T_1_-weighted and FLASH images in each subject were co-registered using an 6-parameter affine transformation in Amira (Visage Imaging) with Lanczos resampling. The two registered FLASH images were summed then smoothed twice using a 3D median filter of kernel size 3 voxels to reduce noise. The filter was applied twice since the kernel size in 3D could not be increased beyond 3 voxels in Amira. The sum of the two co-registered T_1_-weighted images was divided by the filtered, average FLASH image. The T_1_-weighted images and the FLASH images have similar receive field (B^−^) profiles; hence their division produces a ratio image with greatly reduced B^−^ inhomogeneity. The transmit field inhomogeneity (B^+^) is also reduced in the ratio image (Van de Moortele et al., 2009; Marques et al., 2010). Each ratio image was then resampled to 1 mm isotropic resolution and Free Surfer (http://surfer.nmr.mgh.harvard.edu/) (Fischl et al., 2002) was used to extract labels for the cerebrum masked to the pial/CSF boundary. The labels were resampled back to 0.7 mm isotropic resolution to mask the cerebral hemispheres in the original 0.7 mm isotropic resolution ratio image. The subcortical gray matter structures including the basal ganglia and thalamus were manually removed from the masked ratio image, leaving only the cortex and underlying white matter tracts. The ratio image was then segmented into four brain- tissue classes, GM, mGM, WM and CSF (Figure 1). This tissue segmentation was performed with a fuzzy c-means clustering algorithm (Pham and Prince, 1999) in the software package: MIPAV (version 7.0.1, http://mipav.cit.nih.gov/). After segmentation, the two hemispheres were separated for subsequent processing. The output of the fuzzy c-means segmentation was used to produce tissue labels for GM, mGM, and CSF using a fuzzy membership value of 0.5 for those tissue classes. The WM labels were produced using a fuzzy membership level of 0.1. This membership level was used to account for the very thin WM blades in the crowns of the gyrii where WM intensity is reduced due to partial voluming. Finally, in each tissue class, islands unconnected in 3D from the main cluster were removed. The post processing was performed in Amira, unless otherwise stated.

### Morphological measurements

The morphological measurements were performed using the Java Image Science Toolkit (JIST) (www.nitrc.org/projects/jist/) plugins (Bazin et al., 2007; Lucas et al., 2010) v3.0, in MIPAV. The following morphological quantities were measured in each hemisphere (Figure 1).

(1) $$\begin{array}{l} Cortical\;thickness=t\;\\ Thickness\;of\;GM\;in\;the\;outer\;layers\;of\;the\;cortex=d\\ Thickness\;of\;mGM\;in\;the\;deeper\;layers\;in\;the\;cortex=m\; \end{array}$$

For these measurements, level set functions were created for the three boundaries, namely the outer cortical boundary separating CSF and GM or the pial surface, the outer myelinated cortical boundary separating GM and mGM and the outer white matter boundary separating mGM and WM, using JIST implementation of the Distance Field (1.3 R) module in MIPAV.

(2) $$\begin{array}{l} Levelsetfunctionfortheoutercorticalboudary\;=\;\emptyset_{p},\;\\ Levelsetfunctionfortheoutermyelinated\\ corticalboundary\;=\;\emptyset_{m},\\ Levelsetfunctionfortheouterwhitematterboundary=\emptyset_{w} \end{array}$$

The cortical thickness *t* was obtained by subtracting the WM level set function, ∅*_w_*, from the pial surface level set function ∅*_p_*. Similarly, thickness *d* was calculated by subtracting the mGM level set function ∅*_m_* from the pial level set function ∅*_p_*. These thickness calculations were performed using the JIST implementation of the Thickness (1.5 R) module in MIPAV. Next, the difference of these two thickness measurements, *t* and *d* was calculated. For nested surfaces and in regions where strong myelination persists continuously in all the deeper layers of the cortex, measuring the difference *t* − *d* is equivalent to determining the thickness *m* of the strongly myelinated deeper cortex directly as the distance between the outermost edge of the well myelinated tissue and the WM boundary (Equation 3).

(3) m=t-d

Performing all the thickness measurements with reference to the outer GM boundary or pial surface ensured that the thickness data could be displayed on the pial surface.

**Figure 2:**
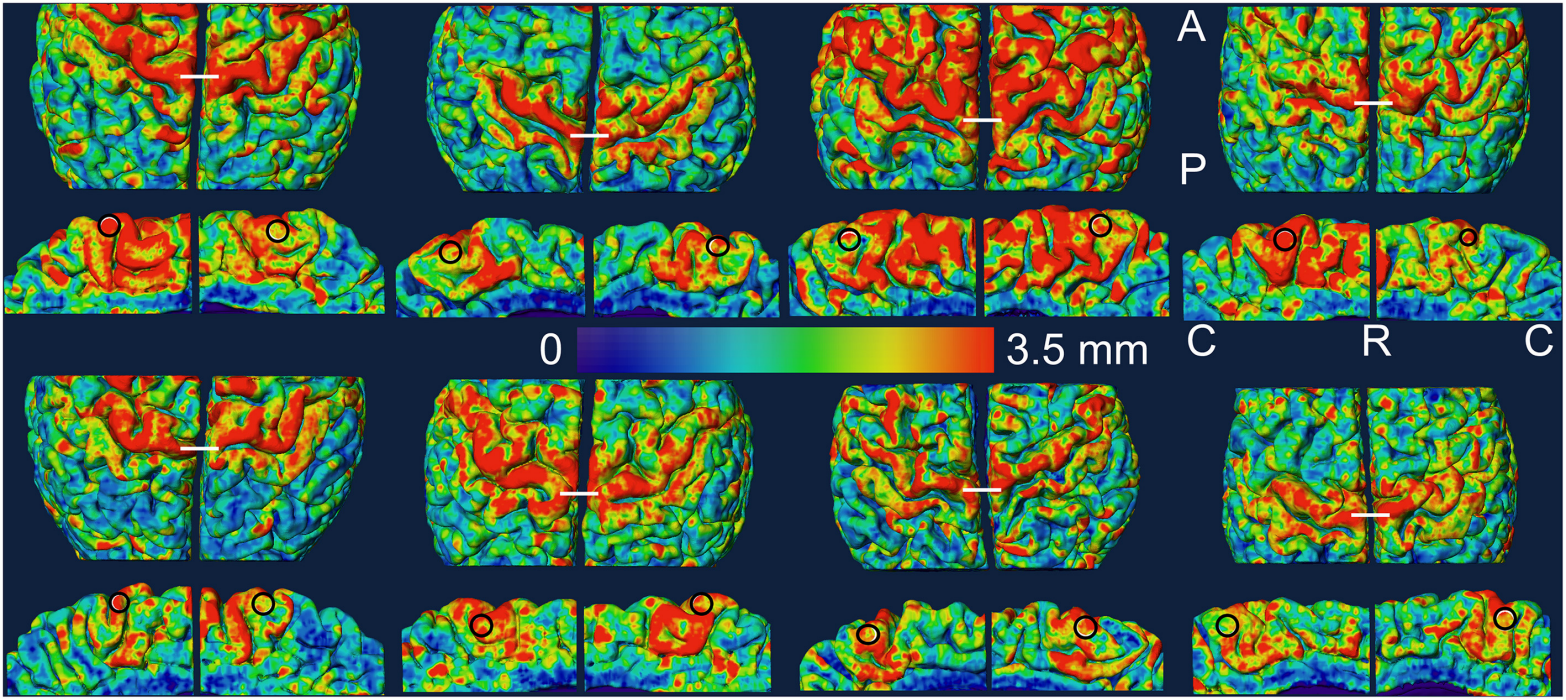
**Myelinated thickness data is projected on the slightly smoothed pial surface in controls (top two rows) and in amputees (bottom two rows)**. For each subject, the medial view of each hemisphere (rows 2 and 4) is displayed under the dorsal view (rows 1 and 3) of the same hemisphere. The left hemisphere is shown on the left in each brain. The ROIs are shown as black circles in the medial views. The white lines in the dorsal views mark the most dorso-medial aspect of the pre-central gyrus. A, anterior; C, caudal; P, posterior; R, rostral.

**Figure 3:**
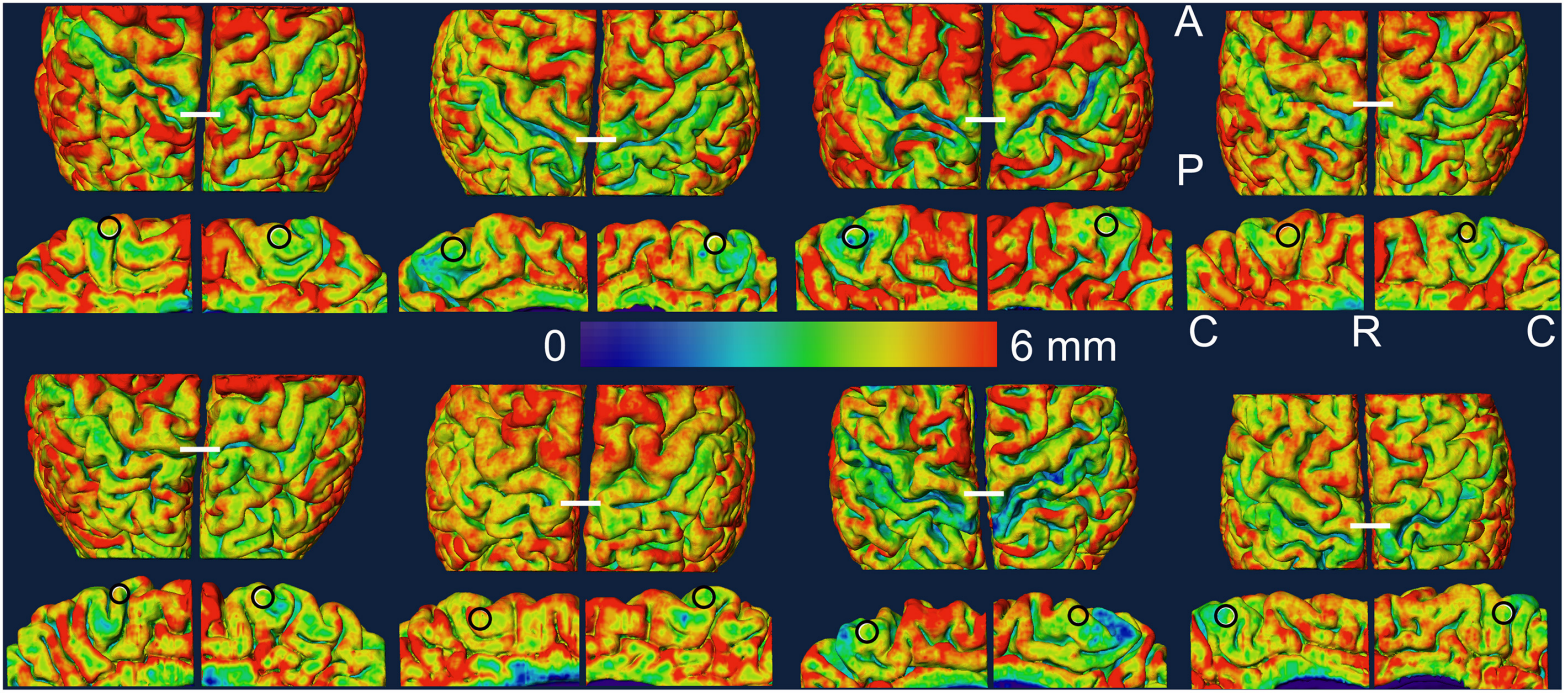
**Cortical thickness data is projected on the slightly smoothed pial surface in controls (top two rows) and in amputees (bottom two rows)**. For each subject, the medial view of each hemisphere (rows 2 and 4) is displayed under the dorsal view (rows 1 and 3) of the same hemisphere. The left hemisphere is shown on the left in each brain. The ROIs are shown as black circles in the medial views. The white lines in the dorsal views mark the most dorso-medial aspect of the precentral gyrus. A, anterior; C, caudal; P, posterior; R, rostral.

The outer cortical boundary was transformed into a triangular mesh to represent the pial surface for visualization and further measurements. The most dorso-medial part of the crown of the pre-central gyrus in the dorsal view was used as a structural marker of the knee representation and the region of interest (ROI) in the lower leg representation was drawn in the medial wall of the hemisphere just below the knee representation. The ROIs were thus hand drawn as approximate circles (area: mean ± STD = 54 ± 4 mm^2^) within the middle one third part of the paracentral lobule close to the superior boundary in each hemisphere using the Surface Editor tools in Amira. The myelinated thickness and cortical thickness data was projected on the pial surface and the thickness measurements were made as the mean value in each ROI. For visualization, the pial surface was slightly smoothed and the thickness data was again projected on the smoothed pial surface. The ROIs were replicated on the smoothed surface for display (Figures 2, 3).

### Inter and intra-group comparisons and statistics

The amputees and the controls were compared on the basis of bilateral differences in the two morphological quantities measured in the study: the myelinated thickness and cortical thickness in the area representing the lower leg in M1. The bilateral difference or bilateral asymmetry in a measured quantity was defined as the absolute inter-hemispheric difference in that quantity divided by its mean inter-hemispheric value. Pair-wise comparisons within each group (left vs. right or affected vs. unaffected) and between the groups (left vs. left and right vs. right) were also performed for both thickness measures. All the comparisons were made using a Kruskal-Wallis test at a significance level of 0.05. Keeping in mind the small sample size, power analysis was also performed, with power = 0.8, to estimate the magnitude of change that would result in statistically significant bilateral differences in the measured quantities with the current sample size of 4 or vice versa.

## Results

In Figures 2, 3, we show the variation of myelinated thickness and cortical thickness respectively, along the slightly smoothed pial surface in all 8 subjects in dorsal and medial views. The ROIs used for the thickness measurements in the lower leg representation in M1 are also shown in the medial views in Figures 2, 3.

Tables 3, 4 summarize the thickness data in controls and amputees respectively. We found a bilateral asymmetry in the myelinated thickness of 17 ± 10%, and 17 ± 5% (mean ± STD over n = 4) in controls and amputees, respectively. For cortical thickness, we found a bilateral asymmetry of 8 ± 6%, and 14 ± 6% (mean ± STD over n = 4) in controls and amputees, respectively. We did not find any statistically significant difference (*p* < 0.05, uncorrected) between the two groups. Using power analysis, we determined that a 125% change in the mean bilateral asymmetry in the myelinated thickness and a 160% change in the mean bilateral asymmetry in cortical thickness in amputees would be statistically significant with the current sample size.

**Table 3:** **Myelinated thickness and cortical thickness measurements in the ROIs in the leg representation in M1 in controls**.

	**Myelinated thickness (mm)**			**Cortical thickness (mm)**		
	**Left**	**Right**	**Asym (%)**	**Left**	**Right**	**Asym (%)**
1	4.0	3.1	25	4.6	4.2	10
2	3.1	3.2	2	3.9	4.2	8
3	2.6	3.1	19	3.4	3.9	15
4	4.1	3.3	22	4.5	4.5	0
Mean ± STD	3.4 ± 0.7	3.2 ± 0.1	17 ± 10	4.1 ± 0.6	4.2 ± 0.2	8 ± 6

*Asym represents bilateral asymmetry. STD represents the standard deviation of the sample. See Supplementary Material (Supplementary Table 1) for STD over each ROI. The subjects are in the same order as in Table 1*.

**Table 4:** **Myelinated thickness and cortical thickness measurements in the ROIs in the leg representation in M1 in amputees**.

	**Myelinated thickness (mm)**			**Cortical thickness (mm)**		
	**Left**	**Right**	**Asym (%)**	**Left**	**Right**	**Asym (%)**
1	3.5	**2.8**	22	4.4	**4.0**	7
2	**3.6**	3.2	12	**4.9**	4.1	18
3	3.5	**3.0**	15	4.4	**3.9**	12
4	**2.9**	2.4	21	**4.1**	3.3	22
Mean ± STD	3.4 ± 0.3	2.8 ± 0.4	17 ± 5	4.4 ± 0.3	3.8 ± 0.4	15 ± 6

*The numbers in bold represent the affected hemisphere contralateral to the amputated limb. Asym represents bilateral asymmetry. STD represents the standard deviation of the sample. See Supplementary Material (Supplementary Table 2) for STD over each ROI. The subjects are in the same order as in Table 1*.

In summary, we observed that bilateral asymmetry in the thickness of the myelinated tissue in the region of M1 representing the lower leg is not significantly altered as a result of long-withstanding lower limb amputation. We also observed no change in bilateral asymmetry in cortical thickness in the region of M1 representing the lower leg in amputees.

## Discussions and conclusion

In this study, we measured myelinated thickness in the bilateral lower leg representations in M1 in four lower limb amputees and four controls. Qualitatively, we observed a similar pattern of variation in the myelinated thickness in the precentral motor cortex in all 16 hemispheres studied.

The results of our morphological measurements of myelinated thickness in the lower leg representation in M1 indicated that bilateral asymmetry was not significantly altered as a result of long withstanding amputation. Also, our results indicate that myelinated thickness was not statistically different between the affected and unaffected hemispheres in the amputees or between the left and right hemispheres in either group. Furthermore, all amputees had a long standing loss of limb which occurred at an early age of 10 years or younger when the brain was still developing (Raznahan et al., 2011). It is therefore very likely that any structural changes that could happen as a result of amputation would have completed their course by the time the amputees were imaged. One short coming of our study was the small sample size. In fact, power analysis of our data indicates that a 2.25-fold bilateral change in the myelinated thickness in the lower leg representation in M1 in amputees would have been required to ensure a statistically significant difference and a larger sample should be included in future studies to investigate if there are more subtle changes in cortical anatomy in amputees. For instance, a modest change of 10% in bilateral asymmetry in the myelinated thickness as measured using our techniques would require 366 subjects. Cortical studies in other cases of sensory deprivation however do not suggest any gross changes in the intracortical myelin patterns in the primary functional areas (Trampel et al., 2011; Voss et al., 2014), and our data corroborates this.

The absence of any statistically observable difference in the morphology of well-myelinated tissue in the lower leg representation in M1 in amputees suggests that the myelination pattern in M1 is not grossly disrupted as a consequence of amputation. The only motor pathway that is affected by amputation is the cortico-spinal pathway descending from the dorso-medial region of the precentral cortex and the upper motor neurons contributing to this tract are the giant Betz cells in layer V of the cortex. These pyramidal cells are known to contribute to the intracortical myelin by means of their well-myelinated axons and axons collaterals (Braitenberg, 1974). Furthermore, it is known that Betz cells in the lower leg area are the largest in size and have the thickest, myelinated axons (Lassek, 1940; Bishop and Smith, 1964). The Betz cells therefore contribute significantly to the myelination in the primary motor cortex and an intact myelination pattern suggests that these Betz cells contributing to the movement of muscles in the previously intact limb remain viable with their myelinated axons intact, when their prescribed output pathway is disrupted.

The viability of these upper motor neurons after amputation can be explained in more than one way. There could be new connections at the cortical level, at the level of spinal cord or at the level of peripheral nerves (Kaas and Qi, 2004). These upper motor neurons could make new connections at the cortical level and join the group of upper motor neurons that synapse with those lower motor neurons in the spinal cord that are innervating the stump or other muscle groups. Or these upper motor neurons could still synapse with the same pool of lower motor neurons that was previously innervating the intact limb but now those lower motor neurons have grown new connections at the spinal cord level or at the peripheral nerve level and are therefore innervating the stump or other muscle groups. A purely morphological study like ours cannot differentiate between these scenarios. In either case however, the viability of these upper motor neurons suggests that they now contribute to the movement of a new group of muscles after amputation. No human anatomical study investigating these scenarios is reported in the literature as yet. There is, however, one non-human primate study that reported no gross changes in the morphology and position of the region of M1 previously dedicated to the now lost limb (Wu and Kaas, 1999).

The potential re-wiring of the still viable upper motor neurons would result in functional reorganization of M1. On a functional level, degeneration of these upper motor neurons would mean absence of motor-task related activation in the deprived cortex and also, failure to excite any muscles (in the stump or elsewhere) by stimulating the deprived cortex. Available data from functional plasticity studies suggest that upper motor neurons stay functionally intact even after deprivation. A recent study in lower limb amputees observed an expansion of activation maps of the stump in M1 of the deafferented hemisphere, spreading laterally to neighboring regions that represent the trunk and upper limbs (Simoes et al., 2012). Another study in upper limb amputees, however, found M1 to be intact with no expansion or lateral shift of the stump muscle representation in M1 (Gagne et al., 2011). Furthermore, an upper limb plasticity study reported sensation of movement in the phantom limb when trans-cranial magnetic stimulation was applied to the deprived motor cortex (Mercier et al., 2006). Non-human primate studies also indicate that movements in other limbs are evoked by intracortical microstimulation throughout the presumed deprived limb region of the contralateral M1 (Kaas and Qi, 2004). Thus, these studies indicate that a rewiring of the contralateral motor cortex happens as a result of limb loss.

This is the first *in vivo* morphological study of the primary motor cortex in amputees. Morphological studies investigating the deprived cortex in other primary functional regions have contradictory results. A small but statistically significant decrease in the total area associated with primary and secondary visual cortices was reported by a human study of the effect of congenital as well as late onset blindness on the visual areas, but the measurement was based on macro-anatomical landmarks rather than on the micro-structure (Park et al., 2009). However, an intact pattern of intracortical myelin was reported by another human study where investigators could detect the stripe of Gennari, a myeloarchitectural hallmark of the primary visual cortex, in congenitally blind people (Trampel et al., 2011). An increase in the intracortical myelin concentration as studied by magnetization transfer ratio MRI is reported by a recent human study in the deprived visual cortex in early or congenitally blind (Voss et al., 2014). Thus, because of insufficient and contradicting data on the features of intracortical myelin in the deprived sensory and motor cortex and due to methodological differences between various studies, our results cannot be compared with the results of previously published studies.

Our results of no statistically significant bilateral difference in the cortical thickness between the two groups also suggest an overall intact morphology in the lower leg representation in M1 after long withstanding amputation. In future studies, it might also be useful to quantify an MRI parameter, such as T_1_, that is correlated to the amount of myelin present in cortical tissue. This would require the use of optimized mapping protocols, such as DESPOT1 HIFI (Deoni, 2007), to account for the high resolution needed to map the parameter over the cortical layers accurately.

We conclude that myeloarchitecture of the primary motor cortex appears grossly intact in lower limb amputees, as investigated with high resolution MRI of the intracortical myelin. Owing to the small sample size, our findings are anecdotal; however, the consistency of the myelinated thickness maps across the subjects suggests that our technique can be used in future plasticity studies in larger groups.

## Conflict of interest statement

The authors declare that the research was conducted in the absence of any commercial or financial relationships that could be construed as a potential conflict of interest.
