# Supplementary material for: Patterns of myeloarchitecture in lower limb amputees: an MRI study
Source: Front Neurosci. 2015 Feb 5;9:15. doi: 10.3389/fnins.2015.00015 (PMC4318335; doi:10.3389/fnins.2015.00015)
Supplement: Supplementary file 1 [file DataSheet1.PDF]

Supplementary Material

Supplementary Table 1: Myelinated thickness and cortical thickness measurements in the ROIs in the leg representation in M1 in controls. Asym represents bilateral asymmetry. STD with each measurement represents the standard deviation over the corresponding ROI. The sample mean and STD are quoted in the bottom-most row.

	Myelinated thickness (mm)			Cortical thickness (mm)		
	Left	Right	Asym (%)	Left	Right	Asym (%)
1	4.0 ± 0.5	3.1 ± 0.3	25	4.6 ± 0.4	4.2 ± 0.3	10
2	3.1 ± 0.5	3.2 ± 0.6	2	3.9 ± 0.5	4.2 ± 0.5	8
3	2.6 ± 0.7	3.1 ± 0.4	19	3.4 ± 0.8	3.9 ± 0.3	15
4	4.1 ± 0.4	3.3 ± 0.5	22	4.5 ± 0.6	4.5 ± 0.4	0
Mean ± STD	3.4 ± 0.7	3.2 ± 0.1	17 ± 10	4.1 ± 0.6	4.2 ± 0.2	8 ± 6

Supplementary Table 2: Myelinated thickness and cortical thickness measurements in the ROIs in the leg representation in M1 in amputees. The numbers in bold represent the affected hemisphere contralateral to the amputated limb. Asym represents bilateral asymmetry. STD with each measurement represents the standard deviation over the corresponding ROI. The sample mean and STD are quoted in the bottom-most row.

	Myelinated thickness (mm)			Cortical thickness (mm)		
	Left	Right	Asym (%)	Left	Right	Asym (%)
1	3.5 ± 0.4	**2.8** ± 0.4	22	4.4 ± 0.4	**4.0** ± 0.4	7
2	**3.6** ± 0.7	3.2 ± 0.5	12	**4.9** ± 0.6	4.1 ± 0.6	18
3	3.5 ± 0.4	**3.0** ± 0.6	15	4.4 ± 0.5	**3.9** ± 0.5	12
4	**2.9** ± 0.4	2.4 ± 0.5	21	**4.1** ± 0.4	3.3 ± 0.5	22
Mean ± STD	3.4 ± 0.3	2.8 ± 0.4	17 ± 5	4.4 ± 0.3	3.8 ± 0.4	15 ± 6
